# Computer-assisted stereoelectroencephalography planning: center-specific priors enhance planning

**DOI:** 10.3389/fneur.2025.1514442

**Published:** 2025-02-27

**Authors:** Debayan Dasgupta, Cameron A. Elliott, Aidan G. O’Keeffe, Roman Rodionov, Kuo Li, Vejay N. Vakharia, Farhan A. Mirza, M. Zubair Tahir, Martin M. Tisdall, Anna Miserocchi, Andrew W. McEvoy, Sebastien Ourselin, Rachel E. Sparks, John S. Duncan

**Affiliations:** ^1^Department of Clinical and Experimental Epilepsy, UCL Queen Square Institute of Neurology, University College London, London, United Kingdom; ^2^Victor Horsley Department of Neurosurgery, National Hospital for Neurology and Neurosurgery, London, United Kingdom; ^3^Division of Neurosurgery, Department of Surgery, University of Alberta, Edmonton, AB, Canada; ^4^School of Mathematical Sciences, University of Nottingham, Nottingham, United Kingdom; ^5^Institute of Epidemiology and Health Care, University College London, London, United Kingdom; ^6^Department of Neurosurgery, Alder Hey Children’s Hospital, Liverpool, United Kingdom; ^7^Department of Neurosurgery, University of Kentucky, Lexington, KY, United States; ^8^UK Comprehensive Epilepsy Program, Kentucky Neuroscience Institute (KNI), University of Kentucky, Lexington, KY, United States; ^9^Department of Pediatric Neurosurgery, Great Ormond Street Hospital for Children, London, United Kingdom; ^10^Developmental Neurosciences Research and Teaching Department, UCL Great Ormond Street Institute of Child Health, London, United Kingdom; ^11^School of Biomedical Engineering & Imaging Sciences, King's College London, London, United Kingdom

**Keywords:** stereoelectroencephalography (SEEG), computer-assisted planning, spatial priors, epilepsy surgery, intracranial EEG, surgical planning

## Abstract

**Objectives:**

This study aims to refine computer-assisted planning (CAP) of SEEG implantations by adding spatial constraints from prior SEEG trajectories (“Priors”) to improve safety and reduce manual adjustments, without increasing planning time.

**Methods:**

Retrospective validation based on 159 previously implanted trajectories (11 cases) planned by the clinical standard CAP and CAP constrained with spatial priors (“CAP + Priors”). Constraints included 31 target and 51 entry zones, created from 98 consecutive patients (763 implanted SEEG trajectories). Each of the 159 previously implanted trajectories was planned by two fellows, once with CAP and once with CAP + Priors, in a randomized order. The time taken to generate the initial computer-generated plan (T1) and the user-edited final plan (T2) were recorded together with the proportions of electrodes that required subsequent adjustments. Clinical implantability was assessed via a blinded review of each trajectory by five independent epilepsy neurosurgeons with expertise in SEEG implantation.

**Results:**

Expert raters considered 88.5% of trajectories implantable, with no difference in acceptability between CAP alone and CAP + Priors (*p* = 0.79). Median (IQR) T1 for CAP to produce complete automated implantation was 4.6 (0.85) min vs. CAP + Priors was 6.3 (2.6) min (*p* = 0.03). There was no significant difference in T2 (time to complete surgeon-edited plan): CAP median (IQR) 105 (22) min, and CAP + Priors median (IQR) 96 (68) min (*p* = 0.92). The CAP + Priors risk score was significantly lower than that for the previously actually implanted trajectories for the 11 plans analyzed (*p* = 0.004), and no different from CAP alone planning. A significant reduction was observed in manual adjustments required with CAP + Priors in the cingulate gyrus.

**Conclusion:**

Using spatial priors from previous implantations enhances SEEG CAP and increases the granularity of trajectory planning. This approach facilitates more standardized planning and allows for the incorporation of experience from multiple expert centers, decreasing the risk of the resultant trajectories and reducing the proportion of trajectories that require manual planning without significantly increasing planning time.

## Introduction

Stereoelectroencephalography (SEEG) is a diagnostic neurosurgical procedure in which multiple depth electrodes are stereotactically implanted in different target regions of the brain to identify the epileptogenic zone (1). SEEG is required in a subset of patients with drug-refractory focal epilepsy (DRFE). SEEG is the most frequently used “stage 2” presurgical intervention when there are discordant non-invasive presurgical data from clinical semiology, scalp EEG, and imaging, or in magnetic resonance imaging (MRI)-negative cases when no lesion is detected (2–4). As with all stereotactic neurosurgical procedures, it is of paramount importance to plan the proposed trajectories precisely and subsequently implement them accurately. The most significant risk in this procedure is intracerebral hemorrhage resulting from damage to intracranial vessels, which results in significant morbidity in 2–3% of cases (5). Given the risk from vascular conflicts, surgeons carefully plan SEEG trajectories to maximize distance from intracranial vasculature while also ensuring accurate targeting of regions of interest (ROIs), avoidance of critical structures, maximizing gray matter sampling, avoidance of other electrodes, and optimal spatial sampling of the putative epileptogenic zone and involved cortical regions, while minimizing intracerebral trajectory length (1). Various computer-assisted planning (CAP) algorithms have been employed worldwide to aid this planning and reduce the time and cognitive load required to produce optimized safe implantation plans (1, 6–8). EpiNav™ is one such planning software that has been used in CAP for SEEG (6, 9–11), tumor biopsy (12), and laser interstitial thermal therapy (13, 14).

We have previously demonstrated that CAP provides faster SEEG planning and improves gray matter sampling, orthogonal drilling angles to the skull, risk scores (optimizing trajectories away from intracerebral vasculature), and intracerebral length using the EpiNav™ planning software (Centre for Medical Image Computing, University College London/King’s College London) when compared to manual planning, particularly with the use of accurate vascular models (11, 15). A pilot study from our group suggested that reference to prior SEEG trajectories through the creation of spatial prior trajectories may enhance CAP (10), allowing computer-assisted plans to be individualized for center-specific preferences while providing all of the advantages of safety metrics and automated planning. This study created spatial priors from 108 electrodes implanted in 12 consecutive SEEG implantation cases and produced 13 entry regions and 14 target regions.

We have built on the above proof of concept to develop and validate the most extensive library of spatial priors, including 763 trajectories from 98 SEEG implantations over 4 years to further refine CAP. This adds the ability to incorporate center-specific and surgeon-specific preferences to CAP-SEEG. This may increase the clinical utility of these advanced planning techniques within expert comprehensive epilepsy surgery centers and allow for learning and collaboration across centers worldwide.

## Materials and methods

### Patient inclusion

In total, 98 patients underwent depth electrode implantation for SEEG as part of their presurgical investigations at The National Hospital for Neurology and Neurosurgery, London, UK, between 2015 and 2019. All patients underwent a standardized expert multi-disciplinary clinical assessment by specialist epilepsy neurologists, neurosurgeons, neuropsychologists, neuropsychiatrists, and neurophysiologists. Electrode implantation schemes, including targets of electrophysiological interest and adjacent eloquent cortex, were created based on an estimation of the likely seizure onset zone synthesized from all preceding presurgical evaluations, including clinical history, semiology, scalp EEG/video telemetry, psychometric evaluations, structural and functional MRI, and positron emission tomography (PET) and single photon emission computed tomography (SPECT) imaging when appropriate.

### Ethical approval

Ethical approval for this study was provided by the Health Research Authority: 12/LO/0377. All patients included in the study provided written consent to the use of the EpiNav™ planning software and for inclusion in research studies.

### Spatial priors library

An extensive SEEG priors library was generated using 763 trajectories from 98 previous implantations at our epilepsy surgery center, defining 31 targets and 51 entry zones from consecutive implantations performed in 2015–2019.

Each of the 731 implanted trajectories was reviewed and categorized into a target and entry region relevant to the classified spatial priors. Each entry or target spatial prior ellipsoid was modeled as a multivariate Gaussian, with the center of the ellipsoid representing the mean of the electrodes categorized by specific targets, such as “anterior cingulate, superior approach.” This means the target is the anterior cingulate gyrus, and the entry ellipsoid corresponds to electrodes that approached this target through a superior trajectory, commonly involving the lateral superior frontal gyrus or the dorsal half of the middle frontal gyrus at our center. The spread of the ellipsoid is then modeled as the standard deviation along the principal components.

**Figure 1:**
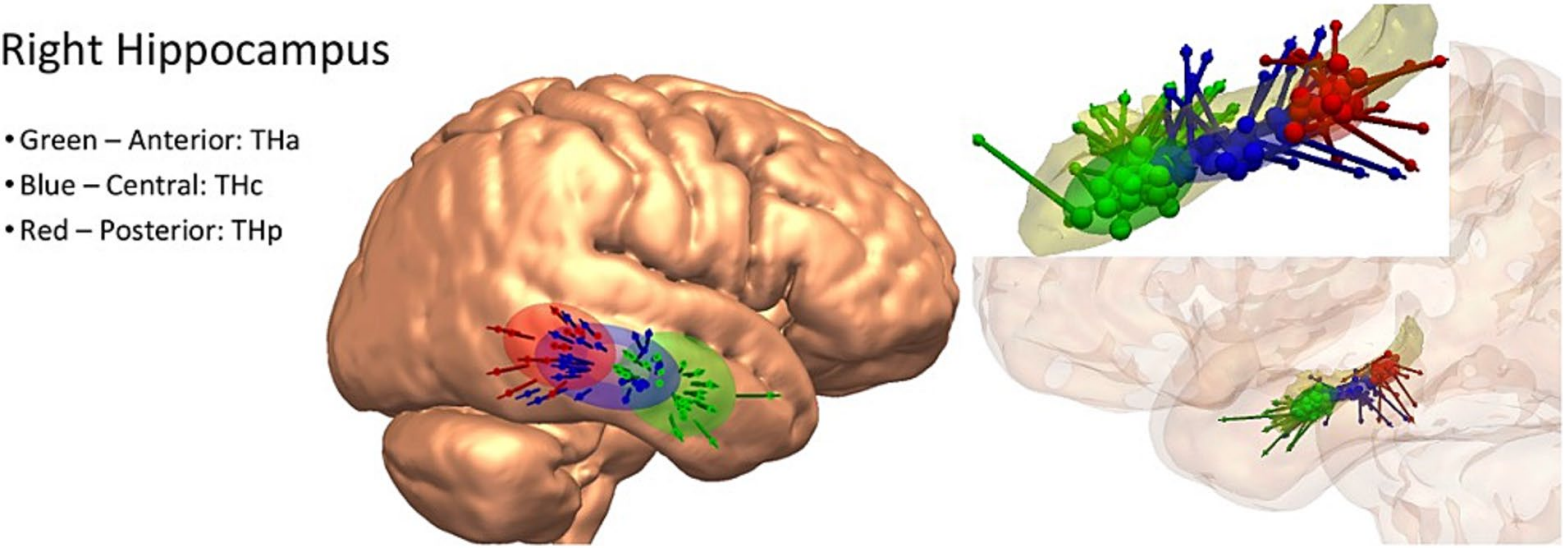
Left: example entry point spatial priors for the right hippocampus, demonstrated on a three-dimensional rendering of the cortical surface of the MNI-152 template brain. Right: example target point spatial priors for the right hippocampus, demonstrated on a three-dimensional model of the hippocampus alone (above) and in the semi-opaque three-dimensional reconstruction of the cortex. Green = anterior hippocampus, blue = central hippocampus, red = posterior hippocampus.

Examples of the created entry and target priors are for the hippocampus in Figure 1 and the anterior insula in Supplementary Figure 1, demonstrating both superior and lateral approaches.

In brief, priors were created as follows:

1 Each electrode was first clustered by its target region (e.g., amygdala and hippocampus).
2 Electrodes placed to target a lesion or patient-specific abnormality were excluded as these are considered “patient-specific”; therefore, they would not be useful for guiding the implantation in a different patient with a different pathology.
3 Each group of electrodes was further subdivided into sub-target regions where there may be several distinct targets (e.g., posterior hippocampus and anterior insula).
4 Finally, each group of electrodes may be further subdivided based on the approach, particularly when there are distinct entry regions (e.g., lateral vs. superior approaches to the posterior insula).
5 Once each group was identified, we:
 a excluded electrodes if they were clear outliers, defined as being outside of 1 standard deviation of the remaining electrodes in that group, and;
 b fitted an ellipsoid to be centered at the mean of the spread of the previously implanted electrodes in the group and having an axis along the three principal directions of variance, with each axis length being 1 standard deviation from the mean (i.e., capturing 95% variance).
6 All electrodes and ellipsoids were reviewed by two consultant neurosurgeons and two senior fellows to verify that the grouping and exclusions were justified and that the resulting ellipsoids were anatomically what would be expected when overlaid on the MNI-152 (ICBM 2009a non-linear asymmetric) group template brain (16, 17) within the three-dimensional rendering of the planning software.

The resultant target and entry priors are described and illustrated in Supplementary Table 1.

### Study design and statistical analysis

The potential benefit of refining CAP by adding SEEG priors (“CAP + Priors”) was measured using a comparison with planning by CAP alone. Planning time, expert raters’ opinions on the implantability of the trajectories, and resultant safety metrics were compared between planning methods, using a dataset of 11 adult SEEG cases at NHNN (159 trajectories; 2016–2020). Each case was planned as per the desired sampling areas set by the clinical multi-disciplinary team (MDT), as done for the actual implantations, and planned twice by two senior fellows working together (see below)—once using the clinical standard CAP and once with the added unit-specific constraints and refinements of “CAP + Priors.” Vascular models for CAP were generated using digital subtraction angiography—the clinical standard at our center (18, 19)—and the images were pre-processed as per the standard clinical planning workflow at our center, previously described in detail (9, 10) and summarized below.

In brief, a gadolinium-enhanced T1-weighted magnetic resonance imaging acquisition is used as a reference image to which all other imaging modalities are registered using NiftyReg (20). A whole brain parcellation is generated using Geodesic Information Flow (GIF) version 3.0 (21), from which automated models of the cortex, sulci, and gray matter are extracted. Vascular models were created following the application of a Sato filter to the digital subtraction angiogram and manual thresholding (22).

Each case was planned by two senior neurosurgical fellows with extensive experience in SEEG planning, working as a team in a randomized order across the 22 plans guided by clinical implantation schematic plans. The times taken for initial computer-generated plan output (T1), user-edited final plan (T2), time spent on each individual electrode, and the proportion of electrodes that required manual planning (having to significantly alter entry and target points from the CAP/CAP + Prior output) vs. minor adjustments following CAP output (such as modifying the position of the tip of the electrode without changing the trajectory: “tip extension”) were all recorded.

In addition, the clinical feasibility of each planning type was assessed by a review of each trajectory by five epilepsy neurosurgeons from external centers who were blinded to the trajectory planning method: CAP alone or CAP + Priors. These ratings were assessed for inter-rater variability using Krippendorff’s Alpha coefficient (*α*) (23), selected because it considers the likelihood of disagreement by chance, making it a measure of both disagreement and non-random disagreements.

Finally, the actually implanted electrodes for the 11 plans of interest were processed through the EpiNav™ pipeline (reconstructing the position of the electrode contact points after coregistering the post-implantation volumetric computed tomography [CT] acquisition), and the safety metrics calculated within the software for each electrode of intracranial length (mm), drilling angle to outer table of the skull (degrees), the risk score [standardized units, a mathematical representation of the size of the avascular corridor through which the electrode passes, described in detail in Vakharia et al. (10)], gray matter:white matter sampling ratio, and minimum distance from vasculature (mm).

The risk score, which we denote by $R\left(\bar{ET}\right)$ , is the primary method of quantitatively assessing and, therefore, allowing comparison of safety between planned electrodes. $R\left(\bar{ET}\right)$ is calculated by sampling 128 evenly spaced points *x* along the planned trajectory $\bar{ET}$ , defined by an entry point *E* and target point *T*, and measuring the shortest distance, *f*(*x*), between the trajectory $\bar{ET}$ and any vessel detected on the preoperative digital subtraction angiography imaging at each node (9, 24). A cumulative score is then provided, scaled by the minimum distance defined by the user $d_{\text{min}}$ (we use 3 mm as standard at our unit) and a maximum distance defined by the user $d_{\text{max}}$ (we use 10 mm as standard at our unit). The 3 mm safety margin or 10 mm risk margin can be altered to the planning surgeons’ preference and is a user-defined setting within the software.


$$R\left(\bar{ET}\right)=\{\begin{array}{c} \overset{128}{\underset{n=1}{\sum }}\frac{d_{\text{max}}-f\left(x\right)}{128*\left(d_{\text{max}}-d_{\text{min}}\right)}\;\mathrm{if}\;f\left(x\right)\geq d_{\text{min}}\forall x\in \bar{ET},\;\\ 1+\overset{128}{\underset{n=1}{\sum }}\frac{d_{\text{min}}-f^{\prime }\left(x\right)}{128*d_{\text{min}}}\;\mathrm{else} \end{array}$$

This equation has the effect of scaling the risk score between 0 and 1 when the trajectory is between $d_{\text{max}}$ (10 mm) and $d_{\text{min}}$ (3 mm), with lower values being farther on average from vessels than higher values. Due to the dense vasculature, we never observed trajectories approximately 10 mm from the closest vessel. When the trajectory falls below the safety margin, we define a capped distance to vessels $f^{\prime }\left(x\right)$ , set equal to 3 mm for any point above the safety margin. This ensures the risk is scored between 1 and 2, with lower values indicating that the trajectory is closer to the safety margin along the entire trajectory length and higher values are closer to the nearest blood vessel.

These metrics were recorded for the electrodes actually implanted in each of the 11 plans of interest (which were historically planned with CAP alone, as is standard practice in our unit), as well as for the synthetic plans using CAP alone and CAP + Priors, as outlined above. These safety metrics were subsequently compared, first comparing the safety metrics of the actually implanted electrodes for these cases against the synthetic CAP + Priors electrodes by exploring the difference between methods using a mixed effects regression model, accounting for possible within-patient clustering (given they were planned on the same patient anatomy and vasculature models).

The mixed effects linear regression models have the form:


$$y_{ij}=\beta_{0}+U_{i}+\varepsilon_{ij}$$

where:

$y_{ij}$ = Difference in outcome (actually implanted measure – CAP + Priors plan measure) for the jth electrode of the ith patient.

$\beta_{0}$ = Estimated difference.

$U_{i}$ = Random effect for the i^th^ patient ( $U_{i}\sim N\left(0,\tau^{2}\right)$ ).

$\varepsilon_{ij}$ = Residual error term ( $\varepsilon_{ij}\sim N\left(0,\sigma^{2}\right)$ ).

The safety metrics for the synthetic CAP alone electrodes were also compared to those for the CAP + Priors electrodes using separate mixed-effects regression models to examine any differences between CAP alone and CAP + Priors planning in the synthetic plans. Statistical analyses were performed using IBM SPSS Statistics© v29 and RStudio. The workflow of the study is summarized in Supplementary Figure 2.

## Results

### Implantability

The clinical implantability of all 318 planned electrodes (159 each planned with CAP alone and with CAP + Priors) was blindly assessed by five expert external epilepsy neurosurgeons. The expert raters determined that 88.5% (487/550) of all trajectories assessed were implantable in their own clinical practice, with no difference in acceptability between the electrodes planned with CAP alone (246/280) and those planned with CAP + Priors (246/277) (Fisher’s exact test *p* = 0.79).

Feedback from the surgeons performing the CAP and CAP + Priors planning suggested that the incorporation of spatial priors constraints into the CAP allowed easier placement of multiple trajectories spaced through large gyri, particularly in the frontal lobe and the superior parietal lobule. It also aided in the visualization of alternative trajectories when manual planning was required, particularly in cases with multiple electrodes in close proximity. Spatial priors, therefore, made SEEG planning easier, particularly when dealing with multiple electrodes in close proximity (avoiding electrode crossing or contact), which is crucial for post-operative MRI imaging and the technical aspects of safe implantation and explantation.

### Planning timings

**Figure 2:**
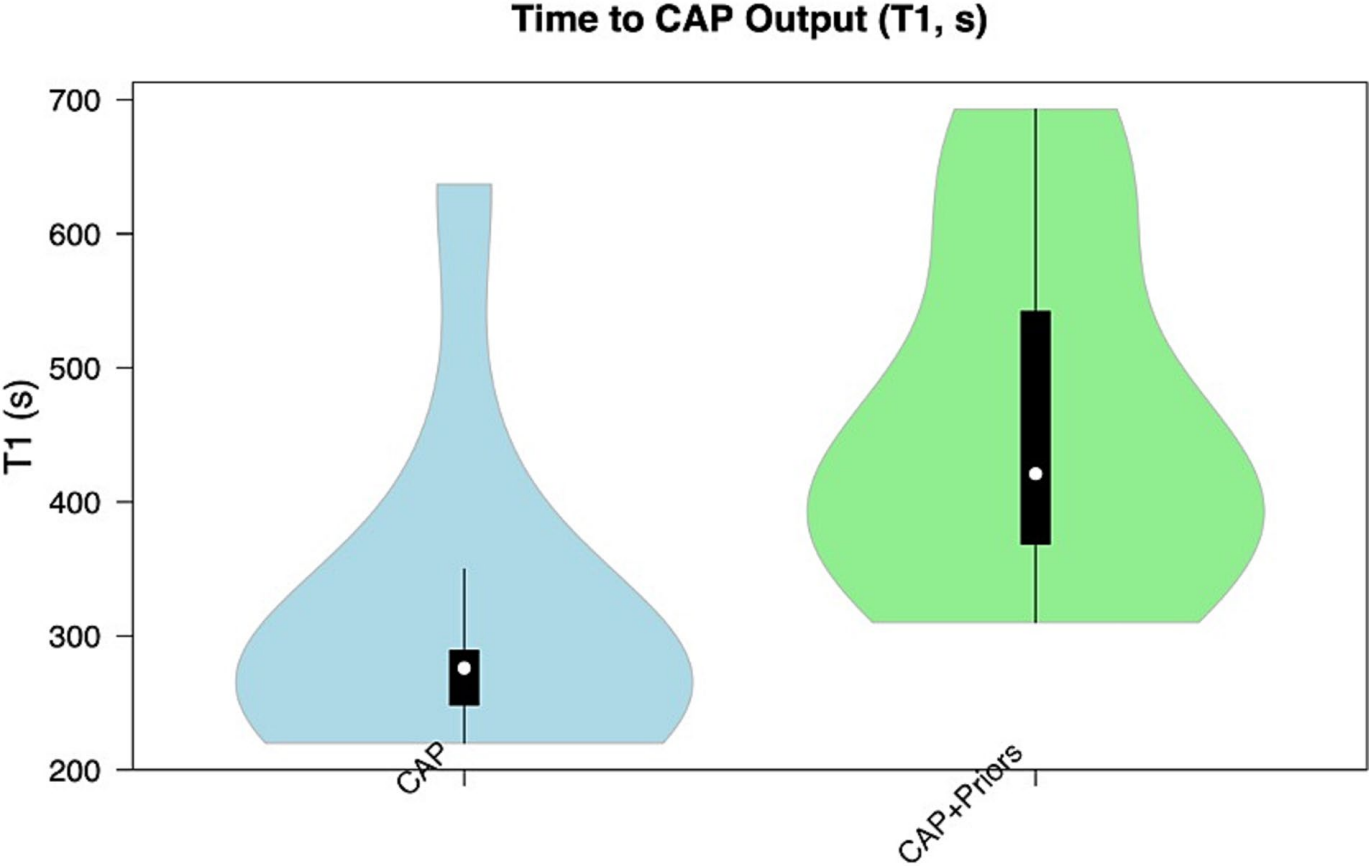
Violin plot demonstrating the distribution of T1 (time to CAP output) in the CAP alone cohort (blue) compared to the CAP + Priors cohort (green).

**Figure 3:**
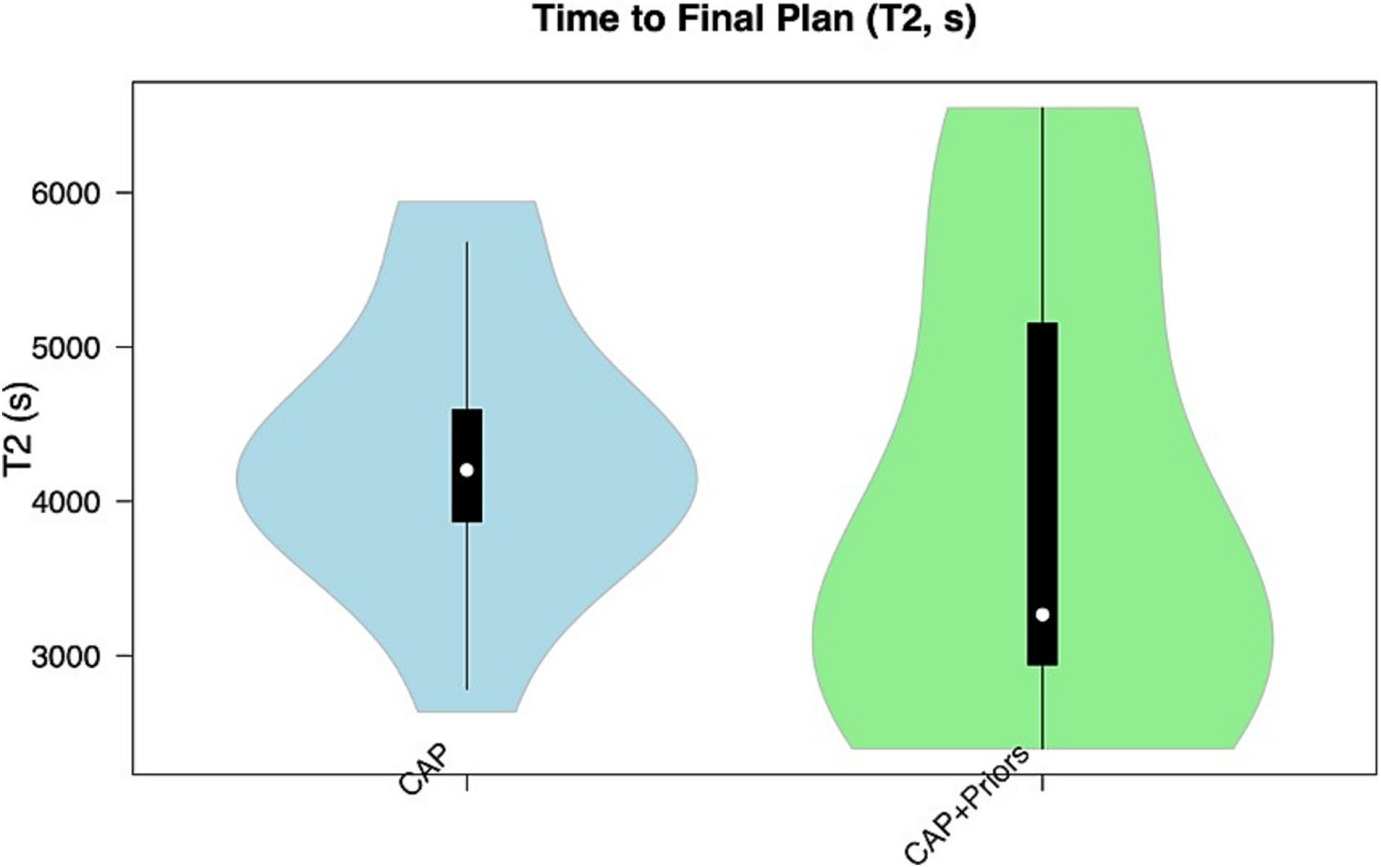
Violin plot demonstrating the distribution of T2 (time to final plan) in the CAP alone cohort (blue) compared to the CAP + Priors cohort (green).

Median T1 (time for initial computer-generated plan output) for CAP alone was 4.6 min (276 s, IQR: 40), vs. CAP + Priors was 7 min (421 s, IQR: 173.5) (*p* = 0.008), a negligible difference in clinical planning time of 2.4 min. Although there was no statistical difference in T2, there was a trend that CAP + Priors planning was, on average, 16 min faster than the final, user-refined implantation plan: CAP median (IQR) was 70 min (12), CAP + Priors was 54 min (37) (*p* = 0.54), a summary of the data is shown in Figures 2, 3.

### Safety metrics

#### Actually implanted electrodes vs. CAP + Priors

The results from multivariate models to compare safety metrics for the actually implanted electrodes against the CAP + Priors electrodes are shown in Table 1. The use of a mixed effects model (accounting for both within-patient and inter-patient clustering) was acceptable, except when the outcome was the drilling angle to the bone, in which case a non-random effects linear regression model was used instead. Model assumptions were checked by exploring residuals, and each model was valid.

**Table 1:** Summary results of a model comparing safety metrics of actually implanted electrodes vs. CAP + Priors planned electrodes.

Outcome	Actual Mean (SD)	CAP + Priors Mean (SD)	Estimated difference (Actual − CAP Priors) and 95% Conf. Int.	Estimate of *τ*^2^	Estimate of *σ*^2^	*p*-Value for a Z-test of *β*_0_ = 0
Length (mm) (*n* = 113)	53.90 (14.96)	55.77 (15.47)	−2.13 (−5.33, 1.06)	14.15	151.06	0.190
Angle (*n* = 113)	19.51 (9.52)	19.49 (9.98)	0.02 (−2.33, 3.36)			0.989
Risk score (*n* = 113)	1.06 (0.10)	1.01 (0.17)	0.046 (0.008, 0.084)	0.001	0.032	0.017*
GWR (*n* = 111)	0.36 (0.18)	0.28 (0.17)	0.080 (0.025, 0.135)	0.010	0.061	0.004*
Min. distance	1.82 (1.09)	2.02 (0.84)	−0.202 (−0.586, 0.182)	0.303	1.19	0.303

Angle = Drilling angle to the outer cortex of the bone, GWR = gray matter:white matter sampling ratio, Min. Distance = minimum distance from blood vessels. *indicates statistically significant difference.

Table 1 shows that there is no statistically significant difference in the length, angle to bone, and minimum distance to blood vessels between the electrodes actually implanted and those planned with CAP + Priors. There is a statistically significant reduction in risk score with CAP + Priors and a decrease in the calculated gray:white matter ratio. This demonstrates not only equivalence but also a reduction in risk score when using CAP + Priors, the primary metric of safety used when planning SEEG implantations.

#### CAP vs. CAP + Priors

Mixed effects models were considered when the difference in the outcome of interest (CAP + Priors method − CAP alone) was modeled, including random effects to account for possible within-patient clustering. However, the inclusion of the random effects was not necessary when estimates were compared to those from standard linear regression models.

Table 2 shows the estimated differences (CAP + Priors − CAP alone) for each outcome of interest, along with 95% confidence intervals and *p*-values for a two-tailed paired *t*-test testing the null hypothesis that the true difference is zero. Model assumptions were checked using standard exploratory plots of residuals and found to be valid. Table 2 demonstrates that the added restrictions and unit-specific limitations introduced by incorporating priors into the CAP process do not adversely affect the safety metrics of the resultant electrodes planned for implantation.

**Table 2:** Summary results of the comparison of safety metrics between the synthetic plans created with CAP alone or with CAP + Priors.

Outcome	CAP + Priors Mean (SD)	CAP Mean (SD)	Estimated difference (CAP + Priors − CAP) (95% CI)	*p*-value (H0: True difference is zero vs. H1: True difference is non-zero)
Length (mm) (*n* = 157)	55.05 (14.37)	53.52 (14.47)	1.53 (−0.25, 3.31)	0.092
Angle (*n* = 157)	19.33 (9.79)	20.51 (11.49)	−1.17 (−3.02, 0.67)	0.211
Risk score (*n* = 157)	1.02 (0.17)	1.02 (0.17)	0.002 (−0.31, 0.03)	0.915
GWR (*n* = 155)	0.29 (0.17)	0.30 (0.14)	−0.02 (−0.04, 0.01)	0.227
Min. distance (*n* = 157)	2.01 (0.84)	2.01 (0.84)	0.004 (−0.15, 0.16)	0.956

Angle = Drilling angle to the outer cortex of the bone, GWR = gray matter:white matter sampling ratio, Min. Distance = minimum distance from blood vessels.

Tables 1, 2 demonstrate that CAP + Priors planning is as safe as CAP alone planning and safer in terms of risk score than electrodes actually implanted without hemorrhagic complications.

### Inter-rater reliability

All five external expert raters’ implantability scores were compared across the four plans that they all rated: Krippendorff’s Alpha coefficient across the five raters and 58 trajectories in four plans, *α* = 0.1982 (95% confidence interval 0.0359–0.3509). This demonstrates very low inter-rater reliability—possible values range from 0 to 1, where 1 is a perfect agreement, and Krippendorff suggests *α* ≥ 0.800 as an acceptable level of inter-rater reliability (25).

This variation reflects the expected variability in practice and preferences for SEEG electrode trajectory planning between practitioners and centers worldwide.

### Reduction in manual planning

The proportion of trajectories requiring subsequent manual planning was reduced from 44.8% in CAP alone to 38.1% when refining the CAP output with priors. However, this difference was not statistically significant (*p* = 0.30). The analysis of the proportion of electrodes requiring manual adjustment following CAP output is summarized in Table 3. This demonstrates a trend that the unedited output electrodes of CAP + Priors appeared to require less manual adjustment than those resulting from the clinical standard CAP alone, particularly for cingulate, frontal, and temporal electrodes, but this was only statistically significant for cingulate electrodes (Fisher’s exact test statistic = 0.0043, significant at *p* < 0.001, using Bonferonni correction for multiple testing).

**Table 3:** Summary of anatomically grouped electrodes comparison against the requirement for manual planning of the automated CAP-assisted electrode trajectory output in CAP alone vs. CAP + Priors.

	Manual adjustment	No significant adjustment required
Temporal electrodes		
CAP	23	32
CAP + Priors	18	38
Insular electrodes		
CAP	13	2
CAP + Priors	9	5
Cingulate electrodes		
CAP	**88**	19*
CAP + Priors	**0**	27*
Frontal electrodes		
CAP	19	25
CAP + Priors	13	31
Parietal electrodes		
CAP	7	10
CAP + Priors	9	8

*indicates statistically significant difference.

## Discussion

This study developed and externally validated an extensive library of spatial priors created from 93 patients to improve CAP-SEEG planning. This allows for the adaptation of CAP-SEEG to incorporate center-specific and surgeon-specific preferences. We demonstrated that planning using the added constraints of previously implanted electrode trajectories grouped to entry and target regions of interest tended to speed up total planning time (Figure 3), building on a previous study showing CAP is faster and safer than manual planning (6). The resultant electrodes are implantable, as judged by five external experts, and safer in terms of risk score when compared to actually implanted electrodes (Table 1). The risk score is the primary method for quantitatively measuring and assessing the proximity of a planned electrode trajectory to the patient-specific intracranial vasculature (6, 24). Therefore, this provides a significant demonstration of safety when compared to actual implanted trajectories, which were demonstrated to be safe as they were implanted without any hemorrhagic complications.

This is a significant step forward in the computer-assisted planning of stereotactic depth electrodes and adds to the suite of techniques to perform this often-time-consuming planning. This technique allows epilepsy surgery centers across the world to adapt their planning using prior trajectories from their own cases and those from other centers, to ensure the output of CAP conforms to their own preferences and techniques, and this does not add significant planning time nor risk to the resultant planned electrodes.

There was a promising trend that CAP + Priors reduced the number of manual adjustments the planning surgeon had to make to the CAP output compared to CAP alone (Table 3). This difference was only statistically significant in the cingulate electrodes; this is likely to have been limited by the number of electrodes assessed across the 11 plans in this study. In addition, it is likely that the priors for the parietal and occipital lobes would benefit from further refinement, as these regions had a comparatively low number of electrodes contributing to the creation of the priors ellipsoids (Supplementary Table 1), as our center implants these regions less frequently than the frontal and temporal lobes.

The implantability of the electrodes planned with CAP + Priors was very high at 88.5% across the five external expert raters (who were blinded to whether the electrodes they were reviewing were planned with CAP alone or CAP + Priors). This is significantly higher than the previous study with external rater validation, in which only 62.2% of CAP-generated trajectories were deemed feasible (11). In addition, there was no difference in implantability between those electrodes planned with CAP alone or CAP + Priors, reinforcing that the raters did not find a negative difference between the groups. This is a strong indicator that CAP + Priors is at least equivalent to our clinical standard of CAP alone.

The low inter-rater reliability shows poor agreement between raters, which is not surprising given the individual preferences that have developed between centers and neurosurgeons. There was a wide range of opinions regarding the perceived safety of the proximity of electrodes to vessels deep in the brain. In contrast, cortical vessels were universally avoided in the planned electrode trajectory.

An example of this variability is the preference for more orthogonal trajectories, which tend to cross sulci deeper in the brain, that may have been born out of using frame-based implantation techniques. In contrast, other surgeons prefer more oblique trajectories that follow the natural directions of the gyri of the brain and avoid crossing the sulci. Given these differing preferences, it is positive that nearly 90% of trajectories were deemed implantable in the practices of the five external experts who reviewed these trajectories.

A limitation of the described technique is that the CAP + Priors approach cannot be used to target patient-specific lesions/anatomical variations, and this is an inherent limitation of computer-assisted planning in general. Electrodes targeting lesions would still need to be manually planned, but these are commonly only a small subset of the electrodes desired in an implantation schema from the multi-disciplinary planning meeting for these implantations. Our experience with implanting lesional cases suggests that using CAP to plan the electrodes not targeting the lesion reduces total planning time and may help tailor the implantation to ensure more cortical coverage of areas of interest. Automating the planning of non-lesional electrodes first allows for a review of any gaps in desired coverage. The planning surgeon can rectify this by subsequently adding manually planned electrodes targeting lesions.

There was a small, but statistically significant, reduction in the ratio of gray matter:white matter sampling in electrodes planned with CAP + Priors compared to those actually implanted (Table 1). This could be interpreted as a negative, however, it is not the amount of gray matter sampling rather the location and relevance of the gray matter sampled to the hypothesis of the epileptogenic zone that is crucial in SEEG as a diagnostic procedure. All of the trajectories are reviewed by the operating surgeon and manually adjusted when required to cover the gray matter of interest, which negates the potential negative here.

The added benefit of using priors is that this allows centers to collaborate and compare their preferences and tendencies, for example, in targeting difficult-to-implant regions, such as the insula, and learn from each other by comparing their prior trajectory entry and target regions and ellipsoids. Such methods could foster learning, collaboration, and discussion between epilepsy neurosurgeons, electrophysiologists, and epilepsy neurologists at comprehensive epilepsy centers worldwide. Indeed, research using these priors is ongoing to analyze similarities and differences in implantations from different centers across adult and pediatric practice, demonstrating the wide applicability of the technique (26). The use of CAP, along with software that allows automated quantification of safety metrics and other metrics for these electrodes across units, also allows a more meaningful and detailed comparison of the differing implantation preferences worldwide.

## Conclusion

This study incorporating external expert review of a comprehensive prior SEEG trajectory library demonstrates that this approach adds to the armory of CAP for SEEG, allows for more granularity of trajectory planning, particularly in large gyri and the hippocampus, and adds a method for including center-specific preferences. The benefit of including spatial priors was particularly evident in the mesial temporal and cingulate electrodes, where the fellows creating the plans noticed a significant difference in ease of planning and reduced time spent adjusting these electrode outputs from CAP + Priors when compared to the output of the clinical standard CAP alone. This technique also allows easier standardization of planning and allows for the future incorporation of experience and expertise from multiple expert centers without significantly increasing planning time and decreasing the risk score (proximity to vessels) of planned trajectories, as well as the proportion of trajectories that require manual planning.

The potential application of this technique, along with the improved granularity of CAP for SEEG across multiple centers, allows for both qualitative and quantitative comparisons of different planning and implantation preferences, offering considerable impact. We envisage the ability to compare and contrast implantation preferences and techniques between expert centers and potentially move toward agreeing to an optimal approach in terms of safety for SEEG implantations with multi-center data. Research building on this and creating further prior libraries to aid in CAP for SEEG implantations is ongoing (26), and it represents an exciting avenue of future study, allowing collaboration between expert comprehensive epilepsy centers worldwide.

## Data Availability

The raw data supporting the conclusion of this article will be made available by the authors upon reasonable request.
