# Supplementary material for: Computer-assisted stereoelectroencephalography planning: center-specific priors enhance planning
Source: Front Neurol. 2025 Feb 27;16:1514442. doi: 10.3389/fneur.2025.1514442 (PMC11905814; doi:10.3389/fneur.2025.1514442)
Supplement: Supplementary file 1 [file Data_Sheet_1.pdf]

Supplementary Table 1

<table>
<tr><th>Prior Name</th><th>Entry Ellipsoid</th><th>Target Ellipsoid</th><th>Number of implanted electrodes used to construct prior</th></tr>
<tr><td>Right-Anterior-Cingulate-Gyrus-superior/lateral-approach (CIAS-L)</td><td rowspan="3">• Yellow – Superior: CIAS-S
• Cyan – Lateral: CIAS-L
• Purple – Inferior: CIAI
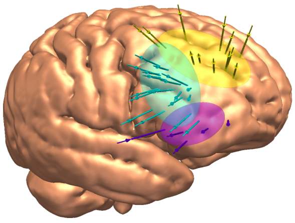</td><td rowspan="3">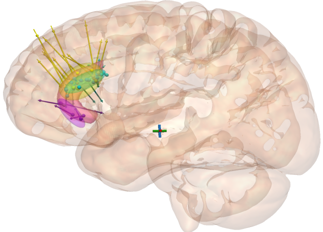</td><td>32</td></tr>
<tr><td>Right-Anterior-Cingulate-Gyrus-superior/superior-approach (CIAS-S)</td><td>23</td></tr>
<tr><td>Right-Anterior-Cingulate-Gyrus/inferior-approach (CIAI)</td><td>10</td></tr>
<tr><td>Right-Middle-Cingulate-Gyrus/lateral-approach (CIC-L)</td><td rowspan="2">• Yellow –Superior: CIC-S
• Cyan –Lateral: CIC-L
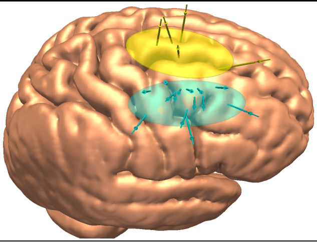</td><td rowspan="2">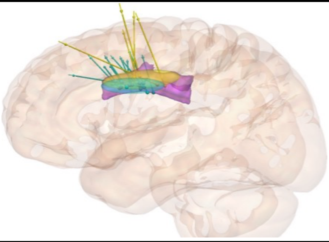</td><td>26</td></tr>
<tr><td>Right-Middle-Cingulate-Gyrus/superior-approach (CIC-S)</td><td>13</td></tr>
<tr><td>Right-Posterior-Cingulate-Gyrus/lateral-approach (CIP-L)</td><td rowspan="2">• Blue – Lateral: CIP-L
• Yellow – Superior: CIP-S
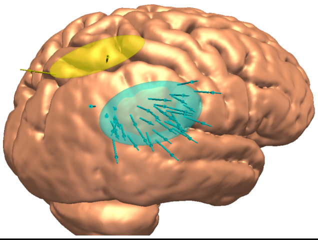</td><td rowspan="2">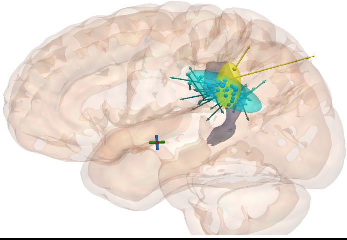</td><td>55</td></tr>
<tr><td>Right-Posterior-Cingulate-Gyrus/superior-approach (CIP-S)</td><td>6</td></tr>
<tr><td>Right-Triangular-part-of-the-Inferior-Frontal-Gyrus (RFIT) OR Right-Pars-Triangularis</td><td>• Green – FIT
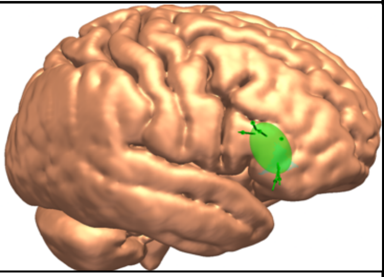</td><td>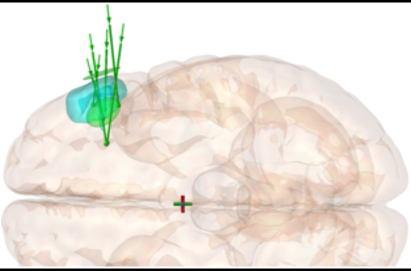</td><td>7</td></tr>
<tr><td>Right-Central-Operculum (COP)</td><td rowspan="2">• Pink– Central Operculum: COP
• Blue – Frontal Operculum: FOP
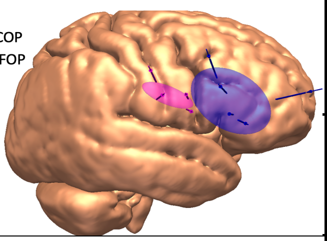</td><td rowspan="2">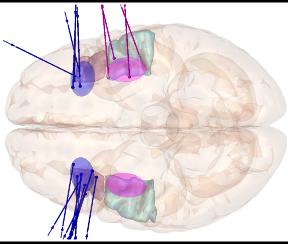</td><td>6</td></tr>
<tr><td>Right-Frontal-Operculum (FOP)</td><td>15</td></tr>
<tr><td>Right-PreCentral-Gyrus-Medial-Segment (FCM)</td><td rowspan="2">• Green – Anterior: FCM
• Red – Posterior : PCM
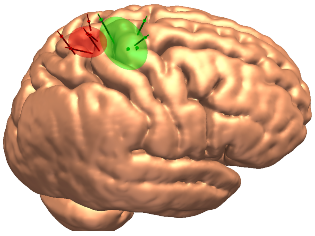</td><td rowspan="2">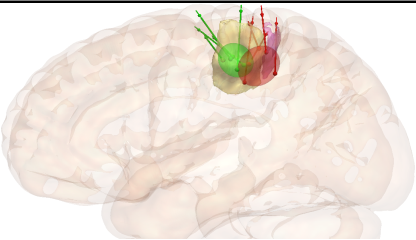</td><td>10</td></tr>
<tr><td>Right-Paracentral-Lobule or Right-PostCentral-Gyrus-Medial-Segment (PCM)</td><td>8</td></tr>
<tr><td>Right-Superior-Frontal-Gyrus-Medial-Segment/lateral-approach (FF-L)</td><td>• Cyan – Lateral: FF-L
• Yellow – Superior: FF-S
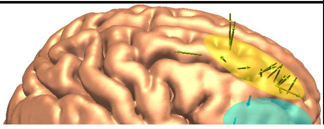</td><td>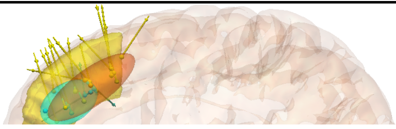</td><td>9</td></tr>
</table>

Right-Superior-Frontal-Gyrus-Medial-Segment/superior-approach (FF-S)	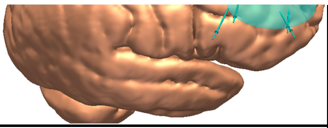	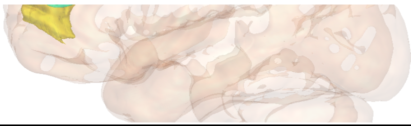	27
Right-Lateral-Orbital-Gyrus/superior-approach<br>or<br>Right-Anterior-Orbital-Gyrus/superior-approach<br>or<br>Right-Posterior-Orbital-Gyrus/superior approach<br>or<br>Right-OrbitalFrontal-Cortex-lateral/superior-approach	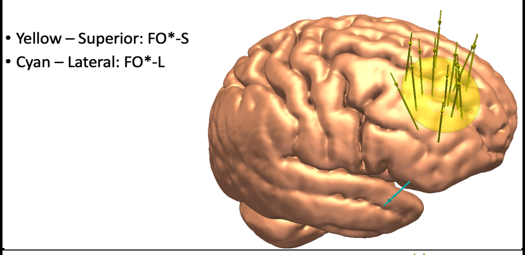	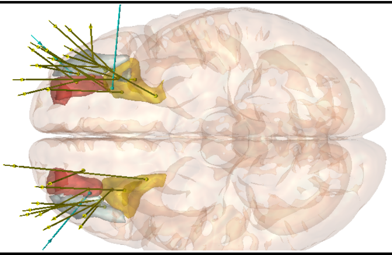	25
Right-Medial-Orbital-Gyrus/lateral-approach<br>or<br>Right-Gyrus-Rectus/lateral-approach<br>or<br>Right-Medial-Frontal-Cortex/lateral-approach<br>or<br>Right-OrbitalFrontal-Cortex-medial/lateral-approach	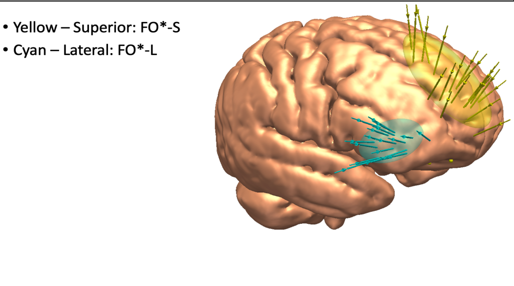	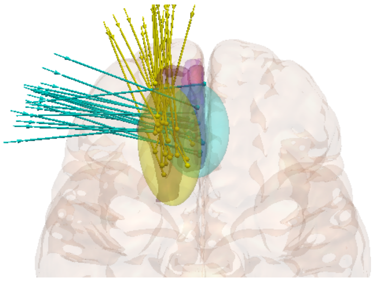	38
Right-Medial-Orbital-Gyrus/superior-approach<br>or<br>Right-OrbitalFrontal-Cortex-medial/superior-approach			34
Right-Supplementary-Motor-Cortex/Area-anterior (FSMA)	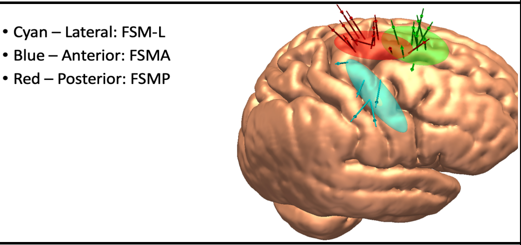	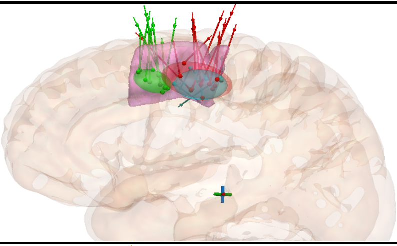	25
Right-Supplementary-Motor-Cortex/Area/lateral-approach (FSM-L)			11
Right-Supplementary-Motor-Cortex/Area-posterior (FSMP)			26
Right-Anterior-Insula/lateral-approach (INA-L)	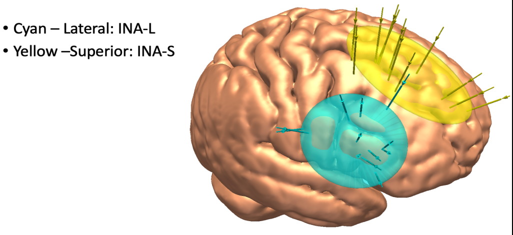	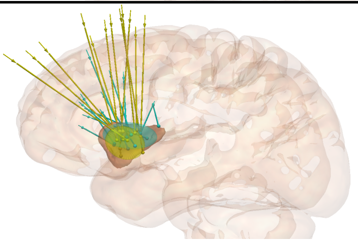	30
Right-Anterior-Insula/superior-approach (INA-S)			22
Right-Posterior-Insula/lateral-approach (INP-L)	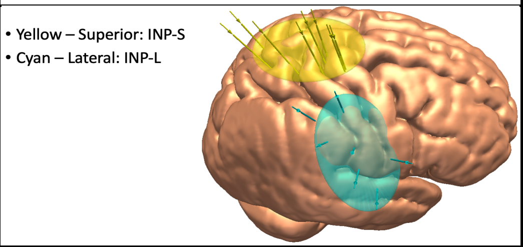	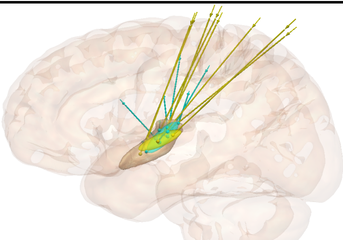	12
Right-Posterior-Insula/superior-approach (INP-S)			14
Right-calcarine-Cortex/lateral-approach (OCA-L)	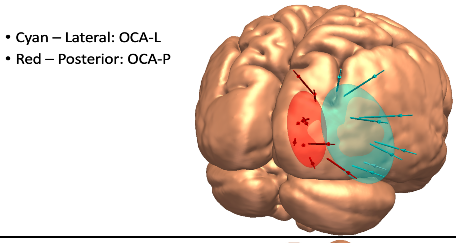	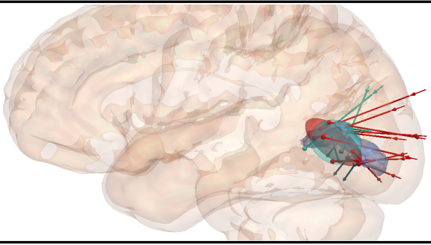	11
Right-calcarine-Cortex/posterior-approach (OCA-P)			11
Right-Cuneus	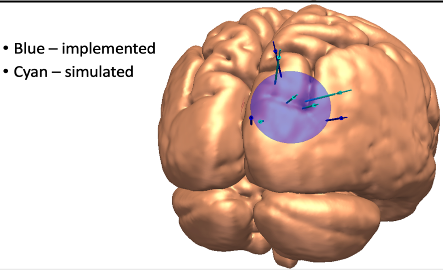	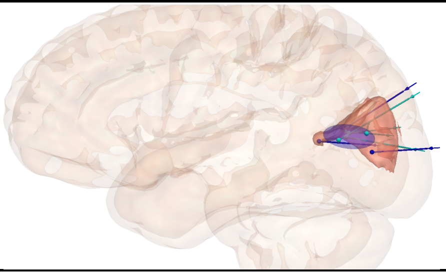	10

Right-Lingual-Gyrus/lateral-approach (OLI-L)	• Cyan – Lateral : OLI-L<br>• Red – Posterior: OLI-P<br>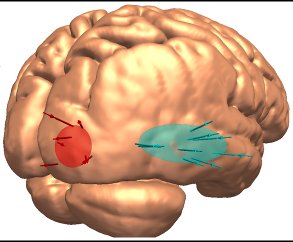	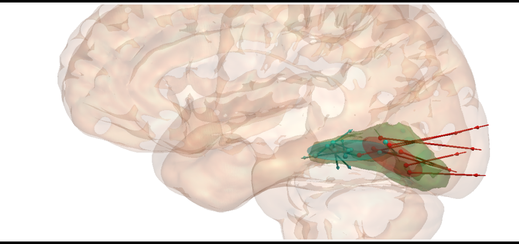	15
Right-Lingual-Gyrus/posterior-approach (OLI-P)			8
Right-PreCuneus/lateral-approach	• Yellow – Superior: PCU-S<br>• Cyan – Lateral: PCU-L<br>• Red – Posterior: PCU-P<br>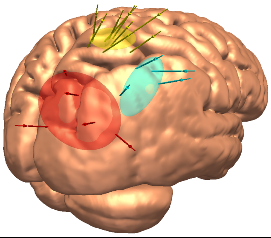	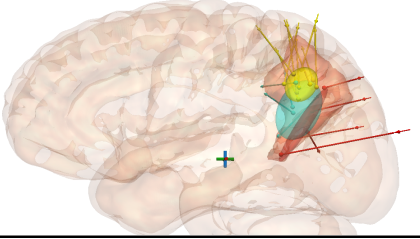	6
Right-PreCuneus/posterior-approach			5
Right-PreCuneus/superior-approach OR Right-Superior-Parietal-Lobule			10
Right-Amygdala	• Red – Amygdala (TAM) / Entorhinal Cortex (TEN)<br>• Green – Temporal Pole (TPO)<br>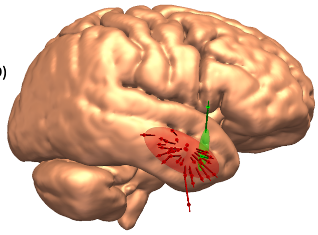	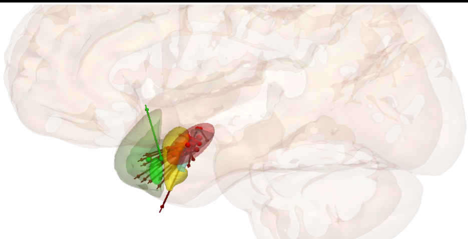	63
Right-Temporal-Pole			5
Right-Fusiform-Gyrus	• Blue – TFU<br>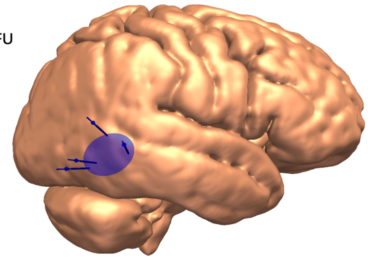	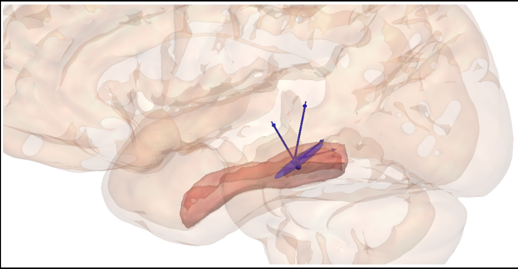	8
Right-Hippocampus-anterior	• Green – Anterior: THa<br>• Blue – Central: THc<br>• Red – Posterior: THp<br>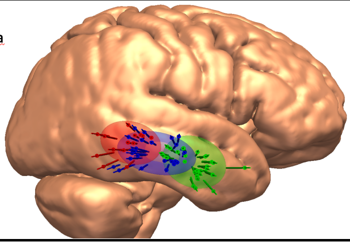	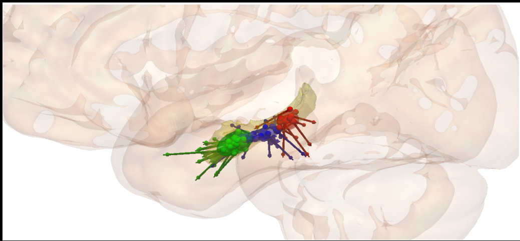	47
Right-Hippocampus-central			44
Right-Hippocampus-posterior			23

Supplementary Table 1: Summary table illustrating the target and entry zone prior trajectories and ellipsoids created to constrain CAP-SEEG planning based on the 763 previously implanted electrodes from 98 SEEG implantations.
