# Supplementary material for: Computer-assisted stereoelectroencephalography planning: center-specific priors enhance planning
Source: Front Neurol. 2025 Feb 27;16:1514442. doi: 10.3389/fneur.2025.1514442 (PMC11905814; doi:10.3389/fneur.2025.1514442)
Supplement: Supplementary file 2 [file Supplementary_file_1.docx]

### Supplementary Figure 1

*
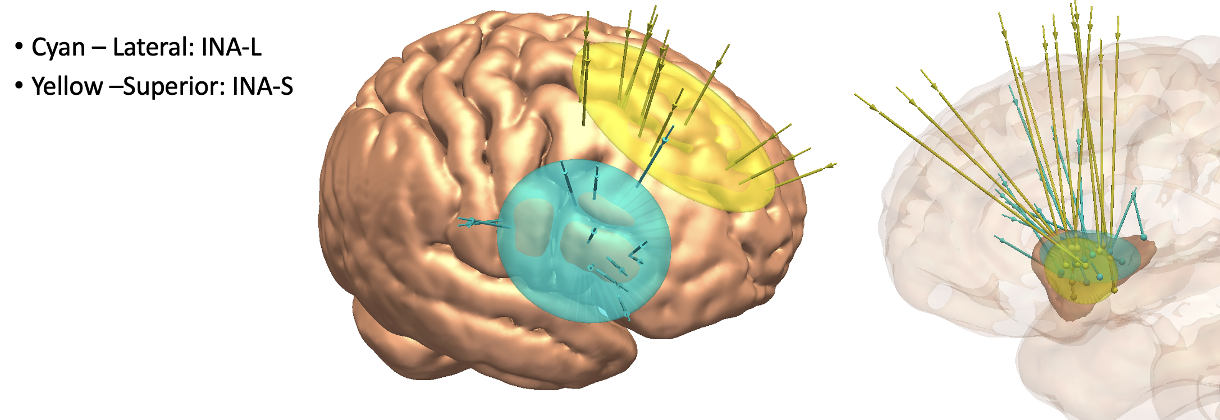
*

*Supplementary Figure 1: : Left – example lateral (cyan) and superior (yellow) entry point spatial priors for the right anterior insula, demonstrated on a 3-dimensional rendering of the cortical surface of the MNI-152 template brain* ^15^*.*

*Right – example target point spatial priors for the right anterior insula, demonstrated on a 3-dimensional model of the semi-opaque 3-dimensional reconstruction of the cortex of the MNI-152 template brain* ^15^*.*

*Cyan = right anterior insula lateral approach entry prior and electrodes, yellow = right anterior insula superior approach entry prior and electrodes, brown (in right image alone) = right anterior insula parcellation derived from Geodesic Information Flow (GIF) version 3.0 ^20^.*

### Supplementary Figure 2


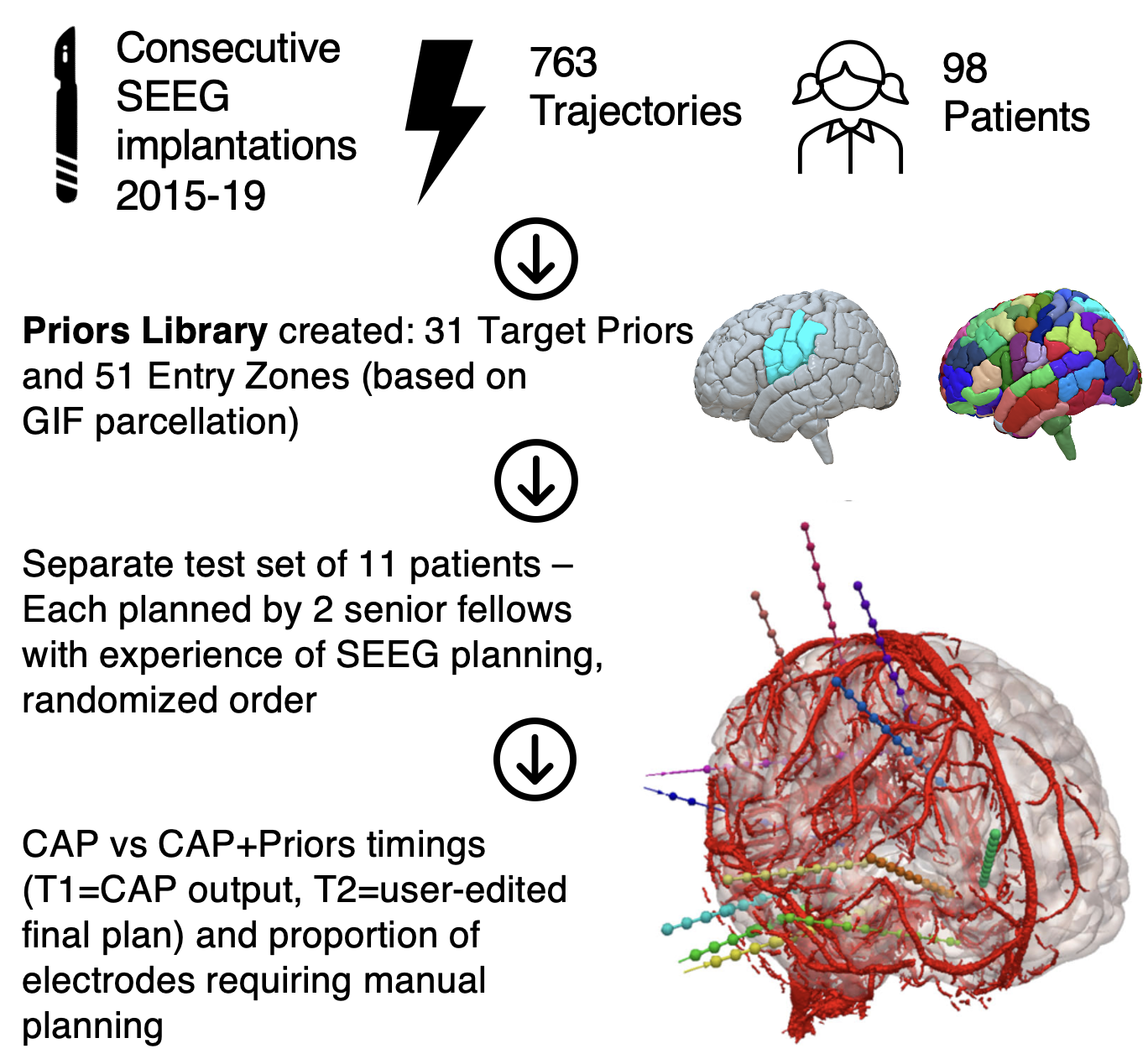


*Supplementary Figure 2: Visual summary of the workflow of this study validating the use of an extensive spatial prior trajectories library to refine computer-assisted planning for SEEG. GIF = Geodesic Information Flow version 3.0* ^20^*.*
